# Dismantling and reimagining global health education

**DOI:** 10.1080/16549716.2022.2131967

**Published:** 2022-10-26

**Authors:** Margaret W. Gichane, Deshira D. Wallace

**Affiliations:** a Department of Obstetrics, Gynecology & Reproductive Sciences, Advancing New Standards in Reproductive Health, University of California, Oakland, OA, USA; b Department of Health Behavior, Gillings School of Global Public Health, University of North Carolina at Chapel Hill, Chapel Hill, NC, USA

**Keywords:** Global health education, colonialism, curriculum, global health workforce, equity

## Abstract

Global health emerged as a distinct public health discipline within the last two decades. With over 95% of Masters of Global Health degree programmes located in high-income countries (HICs), the area of study has been primarily pursued by White, middle and upperclass, citizens of Europe and North America. In turn, the global health workforce and leadership reflect these same demographics. In this article, we present several key arguments against the current state of global health education: (1) admissions criteria favour HIC applicants; (2) the curriculum is developed with the HIC gaze; (3) student practicums can cause unintended harms in low- and middle-income country communities. We argue that global health education in its current form must be dismantled. We conclude with suggestions for how global health education may be reimagined to shift from a space of privilege and colonial practice to a space that recognises the strengths of experiences and knowledge above and beyond those from HICs.

## Introduction

The study of ‘global health,’ is a recent addition to the field of public health education in Europe and North America. In the United States (US), global health emerged as a distinct public health speciality with the first accredited Masters of Global Health degree in 2008 [1]. Since then, the number of programmes has risen rapidly, with now 37 institutions in the US offering masters or doctoral degrees [2]. Universities in Australia, Canada, Kazakhstan, Peru, Rwanda, Sweden, Spain, Taiwan and the United Kingdom also have developed graduate global health degree programmes [3].

The predominance of global health degree programmes in high-income countries (HICs) highlights the imbalance in power in the global health field. Graduates of these programmes often find careers in international organisations and academic institutions, steering global health priorities. Common partners with global health programmes include sites such as the US Agency for International Development (USAID), the World Health Organization (WHO) and the World Bank. Demographic characteristics within these global organisations are not racially and geographically representative. These issues are well documented, with a 1994 report citing ‘protected group members were in many cases underreported by [demographic] category, major occupation, and grade level.’ Even at the partner-country level, foreign service national employees reported that they were ‘professionally underutilised’, indicating a bias towards US employees [4]. A 2020 report indicated that not much has fundamentally changed since 1994 and that USAID continues to struggle with achieving diversity in its ranks which is reflected in a workforce that is 64% White [5]. Similarly, in 2018, an Independent Oversight and Advisory Committee of the WHO found that at WHO headquarters in Geneva, only 25% of staff were from low- and middle-income countries (LMICs). This gap widened at more senior levels of the organisation [6]. A recent report on the governing board seats of global health organisations found that 75% of board seats were held by nationals of HICs and only 2.5% are held by nationals of LMICs. Further, 94% of the institutions were headquartered in HICs [7].

Global health organisations often cite challenges in ‘recruitment of talent’ as a reason for their lack of demographic representation. As education is tied to employment, priorities of these institutions on global health training ultimately inform the future workforce. In the wake of the COVID-19 pandemic, interest in public health careers is at an all-time high in HICs [8]. Given this trend, there is a critical need to reflect and redress how global health degree programmes in HICs exacerbate racial, geographic and class inequities in the health workforce.

## Global health as a pursuit of the privileged

The change in terminology from international to global health was described as shifting from ‘the narrow view of global health as the problems of the world’s poorest societies, to global health as the health of an interdependent global population [9].’ Despite the linguistic shift, global health education in HICs still upholds the colonial legacy tied to international health. Though there are global health programmes in LMICs [10], 95% of masters programmes are in HICs [3]. Thus, conceptualisations of what constitutes global health priorities come from a defined, and skewed, point of view.

Admission eligibility into global health graduate programmes inherently privilege White, middle and upper class, North American and European citizens. The average cost of tuition is nearly US$40,000 [3] not inclusive of programme-related costs and accommodations. These requirements are exclusionary not only due to cost but may also bar individuals due to documentation or citizenship status. Additionally, some programmes [11,12] require or highly encourage a minimum of 1–2 years of experience in a LMIC context. To meet this prerequisite, US-based applicants often complete multi-year volunteer programmes in LMICs such as the Peace Corps, a programme which has been critiqued for its roots in American exceptionalism, White saviorism and imperialism [13]. Further, several global health programmes partner with such organisations for targeted scholarships, thus emphasising the notion that the pathway to global health is through specific organisations deemed as ‘valid.’ Currently, the demographics of the 7,334 Peace Corp volunteers stand as majority women (65%) and only a third identify as racial and/or ethnic minorities [14]. Having strong ties with programmes like the Peace Corps as a pipeline for recruiting future global health leaders suggests that the global health workforce will reflect the demographics and values of these organisations.

The juxtaposition of having explicit and/or implicit requirements to engage in volunteer international work as a means of demonstrating applicant readiness leaves out the experiences of those who live in the countries where volunteers are sent. Learning in an environment built for the privileged can be alienating for those who are viewed to be in lower rungs of the social hierarchy [15]. Their lived experiences are considered illegitimate, even if these applicants have a more nuanced understanding of the feasibility and applicability of projects and programmes in their communities.

## HIC dominant curriculum

Global health education functions as the study of improving health in LMICs by European and North American institutions. Some scholars have defined global health as the study of public health, ‘somewhere else.’[16] A glaring example of this phenomena is the ‘global is local’ debates occurring in US-based Schools of Public Health which are grappling with whether research and practice in the US would constitute global health [17]. This lack of acknowledgement that (1) the US is part of the world and (2) health innovations developed in LMICs would not have utility in HICs is xenophobic. A review of published articles on competencies for global health education found that 12 of 13 articles focused on competencies for students training in HICs [18]. One of the key global health competencies according to the Association of Schools of Public Health is capacity strengthening [19], which is not as widely emphasised in other public health concentrations and inherently assumes a deficit of skills in LMIC regions.

Further, syllabi for global health courses are predominated by HIC-based researchers who are the lead authors on most articles in global health journals [20,21] and textbooks [22]. This focus on Western ways of thinking is an example of epistemic injustice, wherein marginalised groups are not viewed as credible producers, interpreters or recipients of knowledge [23]. While some may feel like simply including more scholarship from LMIC authors would solve this issue, a more critical approach would be to completely reassess the types of knowledges that are valued. What would global health curricula look like if they were built by the communities most affected by health inequities? What format would knowledge be shared? How would learners be assessed, if at all? For global health education to truly be effective, curricula need to reflect the priorities and viewpoints of communities.

## Community harms

Global practicum experiences have the potential to cause immense harm to the institutions and communities where trainees complete them. The short-term practicum experience is one of the corner stones of master’s level public health education. For global health trainees, the required 6–12-week experience is often conducted in an LMIC setting. Whiteness affords power to some HIC trainees, which allows them entrance and a level of credibility not granted to others coded as White and/or coded as ‘American’ or ‘European.’ Whiteness is a racialised socio-political system that economically, socially and ideologically benefits people of European descent, while simultaneously disadvantaging other groups [24]. Institutions have developed pre-departure training to prepare trainees for their practicum experiences outside their home countries. While such trainings are a step towards ensuring trainees are well informed and effective in their practicums, they do have critical gaps. A recent systematic review found that programmes were often short, included limited discussion of ethics, and some had never been evaluated [25]. Students are typically sent to host institutions with just brief training on ‘cultural competency,’ and a cursory understanding of the contexts they will be in. We acknowledge the change from the use of cultural competency (i.e. one can be competent in other cultures) as the framing from training to cultural humility, described as a more life-long reflexive praxis of identities and cultures of self and others [26]. However, even with these shifts, both forms of training are fundamentally ahistorical. What make global health of interest is that projects are executed with little understanding of why and how these health conditions arose in the first place and the role many sending countries have played (and continue to play) in experienced health disparities. Therefore, having training that includes a diverse set of historical texts and perspectives can lead to more informed engagement with communities

Most research examining the harm inflicted in global health training has primarily focused on prospective and current medical school trainees. Ethical violations include conducting procedures beyond their current training, limited oversight of the care provided, lack of follow-up or even data to evaluate the outcomes of these short-term medical missions [27–29]. However, non-clinical global health students are still culpable in perpetuating the same violations. With limited experience in the host country, trainees may focus on topics that are not aligned with community priorities and may offer ‘solutions’ that are not feasible, sustainable or needed in the setting [28]. Feedback from host institutions indicate that students coming from HICs lack sensitivity to cultural norms and leave with little follow-up [30]. These actions may waste precious time and resources and damage the relationship between the host institution and their surrounding community.

## Conclusion

A degree programme which seeks to predominantly train students from HICs to ‘solve’ the health problems of LMICs is a modern reinvention of The White Man’s Burden and still carries the legacies of colonial and tropical medicine which came before [31]. We propose that global health education, as is currently imagined, should be dismantled. We implore global health programmes to critically question who they serve and how they contribute to inequity. Global health programmes create a positive feedback loop where larger national and multinational organisations construct the priorities of what is to be studied and where. This leads to systematic exclusion of individuals from countries that are often the subject of global health policies and missions. Global health programmes can interrupt this harmful feedback loop.

We acknowledge that the rise of global health programmes means that they are not going away, but they can be reimagined to shift from a space of privilege and colonial practice to a space that recognises the strengths of experiences and knowledge above and beyond those from HICs and even with those countries, from White communities.

A few recommendations for change for these programmes are

- To move away from “global health” being a study of the privileged, HIC institutions need to engage in several strategies. First, schools need to be more transparent on who is currently represented in these programmes. In researching for this piece, we found that schools and programmes that had global health concentrations did not report any demographic information on their students. While privacy may be a concern, aggregating these numbers would allow for transparency into what the potential workforce looks like. It also increases accountability for these programmes to improve recruitment and retention of students from LMICs and those historically and contemporarily excluded from educational opportunities. Next, institutions need to work towards making global health education in HIC more accessible to LMIC students. HIC programmes need to ensure that LMIC applicants who wish to study in HIC are provided with adequate funding to cover tuition and other programme expenses. During the pandemic, some HIC institutions have used online teaching to bring in experts and trainees from around the world [32]. Such opportunities should come with significant honorariums and opportunities to earn credentials for LMIC students. Finally, and most importantly, HIC institutions must make investments in training in LMIC institutions. Academics in HIC institutions hold significant power in global health organisations and funding bodies. Using their power to channel funding to LMIC institutions would have the most lasting impact on the field.
- The epistemic injustice framework developed by Bhakuni and Abimbola [23] offers a tool to critically examine how knowledge is produced in global health and for whom. Using this framework in the development of curricula may help decentre HIC voices. Curricula should shift from abstract notions of “culture” during training and focus on histories of countries, regions and peoples that students, practitioners and researchers work with. These histories need to come in a variety of modes (non-fiction, fiction, memories, poems, audio and visual media) and need to primarily focus on individuals from these countries.
- Institutions with global health programmes need to audit programme requirements to evaluate if they present an undue burden on prospective and current students. This includes what is expected of summer practicums. Vlein and colleagues outline a checklist of guiding questions institutions can implement to decide whether a global health trip meets ethical standards, is equitable between sending and host institution, and does not pose an environmental burden [33]. In addition to undue burden, requirements need to be evaluated to determine if they contribute to structural advantages for privileged students who can engage in a variety of activities which make them more likely to gain access to future jobs.

The journey to dismantle and reimagine global health education is long overdue. We join in solidarity with current efforts to redistribute power and decentre HICs in global health education [34,35].

## Paper context

Power in global health leadership, research, and practice has been primarily held by institutions in high-income countries. In a moment where institutions are being asked to decolonise, an area that has received less attention is global health education. Our paper provides critique of who is enrolled in global health degrees, what is taught in global health curricula, and community harms. We offer a set of solutions on how global health education can be reimagined.
